# Dietary selection of commensal protists: expanding host immunity by impacting trans-kindom competition with bacteria

**DOI:** 10.1038/s41392-024-01834-z

**Published:** 2024-05-06

**Authors:** Bo Li, Min Wu, Huaqiong Li

**Affiliations:** 1 Department of Neonatology, The Second Affliated Hospital of Wenzhou Medical University and Yuying Children’s Hospital, Wenzhou Medical University, Wenzhou, Zhejiang 325027 China; 2 Zhejiang Engineering Research Center for Tissue Repair Materials, Wenzhou Institute, University of Chinese Academy of Sciences, Wenzhou, Zhejiang 325000 China

**Keywords:** Immunology, Microbiology

In a recent publication in *Cell*,^1^ Elias R. Gerrick and colleagues identified novel commensal parabasalid protists (i.e. *T. casperi, Parabasalia_sp_1653*) with distinct metabolic traits, including diverse succinate excretion and dietary preference, which drives different trans-kingdom competition with specific commensal bacteria and results in divergent small intestine (SI) immune response, highlighting the significance of dietary interventions as a pivotal strategy for maintaining gut health (Fig. 1).

**Fig. 1:**
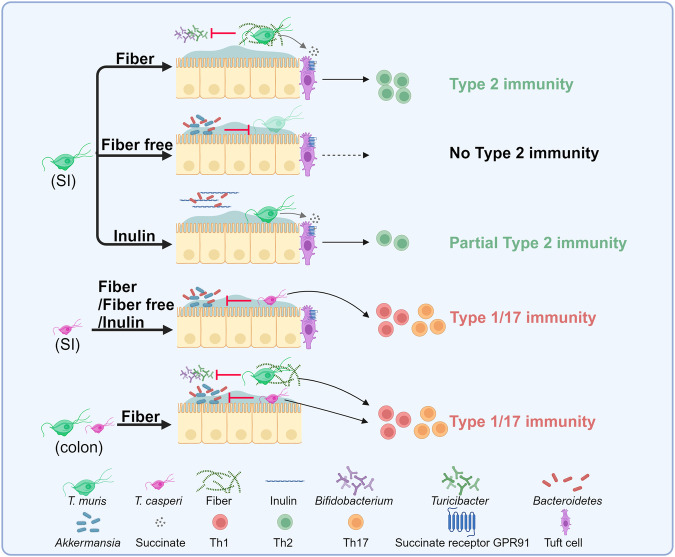
Illustration of metabolic diversity within protists and how dietary composition influences the host’s immune response. Graphic adapted from the graphical abstract of “Metabolic diversity in commensal protists regulates intestinal immunity and trans-kingdom competition”.^1^ *T. muris* (*Tmu*) and *T. caspri* (*Tc*) elicit similar type 1/17 immune response in the colon but divergent type 2 immunity in the small intestine (SI). During dietary fiber starvation, *Tmu* shifts to a mucolytic metabolism but encounters substantial competition from mucolytic bacteria, such as *Bacteroidetes* and *Akkermansia muciniphila.* However, with inulin as the exclusive dietary fiber, *Bacteroidetes* species capable of fermenting both inulin and mucus reduce the competitiveness with *Tmu* for mucus glycans, leading to moderate type 2 immune responses. (Created with BioRender.com)

The gut microbiota is crucial for initiating and sustaining the functions of the host’s immune systems. Imbalance within the gut microbiota can lead to a spectrum of severe health issues, including inflammatory bowel diseases (IBD), allergies, and metabolic syndromes, thereby positioning the microbiome management as a critical therapeutic strategy for these conditions. For instance, the oral administration of bacterial strains known to induce CD4^+^FOXP3^+^ regulatory T (T_reg_) cells in humans to mice has been shown to mitigate symptoms of colitis and allergic diarrhea.^2^ Additionally, dietary choices can also impact the metabolism and composition of the microbiota, subsequently influencing disease progression or symptom alleviation.^3^ Although the majority of microbiome research focuses on bacteria due to their abundance, the ecosystem also encompasses fungi, archaea, viruses, and protists. The contributions of commensal protists to host health and immune system regulation are often underappreciated, given the common perception of protists as primarily pathogenic. Commensal protists, particularly those from the *Parabasalia* phylum, coexist with humans and animals across diverse settings, and play a significant role in shaping intestinal immunity in mice.^4^ Considering their prospection in treating intestinal diseases, it is imperative to elucidate the fundamental characteristics of commensal protists.

*D. fragilis*, the predominant Parabasalid in industrialized populations, has been ambiguously classified because of its occurrence in both symptomatic and asymptomatic individuals.^5^ Although *D. fragilis* is associated with irritable bowel syndrome (IBS), it intriguingly also appears to offer protection against IBD flare-ups, suggesting that its presence might reduce the risk of IBD. Within gut environments, how *D. fragilis* impacts the host’s immune system and overall health remains largely unknown. The scarcity of appropriate laboratory animal models further constrained our comprehension of *D. fragilis*’s clinical relevance. Considering that murine *Tritrichomonas musculis* (*Tmu*) shares the same phylogenetic order as *D. fragilis*,^1^ investigating the interactions between *Tmu* and the murine intestinal immune system represents a valuable approach for elucidating *D. fragilis*’s role in the human gut immunity. Amongst the gut microbiota, certain microbial species independently exert a significant impact on the immune system. For instance, by promoting the proliferation of T helper 1 (Th1) and T helper 17 (Th17) cells in the SI, Segmented filamentous bacteria (SFB) play a key role in strengthening the mucosal immune system.

Studies also highlight the significant impact of *Tmu* on the colonic immune response in the mice it colonizes.^4^ *Tmu* stimulates Th1 and Th17 immunity through IL-18, which is triggered by the host’s epithelial inflammasome, thereby offering substantial protection against mucosal bacterial infections.^4^ However, *Tmu* colonization in mice has also been associated with increased risk of inflammatory diseases and cancer, aligning with research indicating that the IL-18 receptor may elevate the risk of IBD and cancer in humans. The exploration of *Tmu*’s impact on the host immune system and its potential role in promoting intestinal diseases and cancer are critically important. Parabasalid protists, including *Tmu*, utilize two fermentation pathways: one converting pyruvate to lactate and the other transforming malate to succinate, both of which are excreted as waste. Notably, succinate specifically triggers type 2 immunity within the SI. The intricate fermentation pathways and metabolic impacts of *Tmu* on the host immune system warrant further comprehensive investigation.

Gerrick and his colleagues identified a previously unrecognized commensal *Tritrichomonas* species, *T. casperi (Tc)* in mice and two novel parabasalid species in humans, closely related to *Tmu* and *Tc*, revealing the extensive diversity and widespread occurrence of commensal protists in both mice and humans. Both of *Tmu* and *Tc* trigger similar Th1 and Th17 immune responses in the colon via inflammasome. However, they drive distinct type 2 immune responses by stimulating the succinate receptor GPR91 located in tuft cells of the SI, owing to their distinct metabolic processes, including succinate excretion. While *Tmu* exclusively utilizes the succinate-production pathway, *Tc* produces both succinate and lactate but does not release sufficient succinate into the intestinal lumen to trigger type 2 immunity. Collectively, these findings illuminate metabolic differences in *Tmu* and *Tc* that drive similar Th1/Th17 immunity in the colon, but different Th2 immunity in the SI, which may broaden the research scope and significance with comparative immune system research.

Gerrick et al. further explored whether dietary inputs influence immune response in the SI. By altering the fiber content in mouse diets, the authors observed *Tmu*’s preference for dietary microbiota accessible carbohydrates (MACs), outcompeting significant fiber-degrading bacteria like *Bifidobacterium pseudolongum* and *Turicibacter sanguinis*. In contrast, *Tc* depends on host-derived carbohydrates, outcompeting the mucin specialist *Akkermansia muciniphila*. Analysis of carbohydrate-active enzymes (CAZymes) confirmed the competitive nutritional dynamics between these commensal parabasalids and bacteria specializing in fiber or mucus digestion, demonstrating the trans-kingdom competition between them. Intriguingly, *Tmu* may also ferment mucus glycans, as evidenced by the expression of mucolytic CAZymes. Despite a successful metabolic shift towards fermenting mucosal sugars in their own created medium, parabasalid biphasic formulation (PBF), the investigators also found that *Tmu*’s survival in vivo was challenged under dietary MAC deprivation, largely outcompeted by the mucolytic bacteria such as *Bacteroidetes spp*. and *A. muciniphila*. Moreover, with inulin as the exclusive dietary fiber, *Bacteroidetes* species capable of fermenting both inulin and mucus reduce the competition with *Tmu* for mucus glycans, facilitating a moderate type 2 immune response. These findings reveal that altering dietary fiber consumption significantly impacts the host immune response, particularly inducing graded type 2 immune responses modulated by *Tmu*.

Dietary choice significantly influences the host’s immune systems and health condition, by regulating the microbiome components through trans-kingdom competition. Gerrick et al. introduced a novel medium, PBF, which facilitated the prolonged cultivation of *Tmu*, along with valuable tools and datasets for the exploration of commensal tritrichomonads, paving the way for overcoming previously-insurmountable obstacles to understanding these protists’ immune mechanisms. With such effective research methods, the authors unravel the complex interplay between diet and host’s immunity. Understanding how specific dietary choices affect commensal protists and the host immune system may lead to new theories and strategies for enhancing human health.

## References

[1] Gerrick ER (2024). Metabolic diversity in commensal protists regulates intestinal immunity and trans-kingdom competition. Cell.

[2] Atarashi K (2013). Treg induction by a rationally selected mixture of Clostridia strains from the human microbiota. Nature.

[3] Wastyk HC (2021). Gut-microbiota-targeted diets modulate human immune status. Cell.

[4] Chudnovskiy A (2016). Host-Protozoan Interactions Protect from Mucosal Infections through Activation of the Inflammasome. Cell.

[5] Stark D, Barratt J, Chan D, Ellis JT (2016). Dientamoeba fragilis, the Neglected Trichomonad of the Human Bowel. Clin. Microbiol. Rev..

